# Raman Nanotags‐Guided Intraoperative Sentinel Lymph Nodes Precise Location with Minimal Invasion

**DOI:** 10.1002/advs.202102405

**Published:** 2021-11-05

**Authors:** Binge Deng, Yaohui Wang, Yifan Wu, Wenjin Yin, Jinsong Lu, Jian Ye

**Affiliations:** ^1^ State Key Laboratory of Oncogenes and Related Genes School of Biomedical Engineering Shanghai Jiao Tong University Shanghai 200030 P. R. China; ^2^ Department of Breast Surgery Renji Hospital School of Medicine Shanghai Jiao Tong University Shanghai 200127 P. R. China; ^3^ Shanghai Key Laboratory of Gynecologic Oncology Renji Hospital School of Medicine Shanghai Jiao Tong University Shanghai 200127 P. R. China; ^4^ Institute of Medical Robotics Shanghai Jiao Tong University Shanghai 200240 P. R. China

**Keywords:** biotoxicity evaluations, gap‐enhanced Raman tags (GERTs), sentinel lymph node (SLN), surgery windows, tracer

## Abstract

The accurate positioning of sentinel lymph node (SLN) by tracers during surgery is an important prerequisite for SLN biopsy. A major problem of traditional tracers in SLN biopsy is the short surgery window due to the fast diffusion of tracers through the lymphatics, resulting in a misjudgment between SLN and second echelon lymph node (2^nd^ LN). Here, a nontoxic Raman nanoparticle tracer, termed gap‐enhanced Raman tags (GERTs), for the accurate intraoperative positioning of SLNs with a sufficient surgical time window is designed. In white New Zealand rabbit models, GERTs enable precise identification of SLNs within 10 min, as well as provide the surgeon with a more than 4 h time window to differentiate SLN and 2^nd^ LN. In addition, the ultrahigh sensitivity of GERTs (detection limit is 0.5 × 10^−12^ m) allows detection of labeled SLNs before surgery, thereby providing preoperative positioning information for minimally invasive surgery. Comprehensive biosafety evaluations carried out in the context of the Food and Drug Administration and International Standard Organization demonstrate no significant toxicity of GERTs, which supports a promising clinical translation opportunity of GERTs for precise SLN identification in breast cancer.

## Graphical abstract

Nontoxic gap‐enhanced Raman tags (GERTs) are developed for accurate intraoperative identification of sentinel lymph node (SLNs) within 10 min in rabbit models and with a more than 4 h time window to differentiate SLNs and second echelon lymph nodes (2^nd^ LNs). The ultrahigh sensitivity of GERTs allows detection of labeled SLNs before surgery, thereby providing preoperative positioning information for minimally invasive surgery.

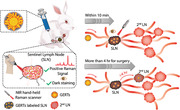

## 1 Introduction

Sentinel lymph nodes (SLNs) are the first regional lymph nodes (LNs) with primary tumor metastasis. Nowadays, SLN biopsy plays a pivotal role in assessing disease progression and influencing therapeutic decision‐making, so there is a growing body of literature that recommends it for breast cancer,^[^ ^1^ ^]^ melanoma,^[^ ^2^ ^]^ gynecological cancer,^[^ ^3^ ^]^ prostate cancer,^[^ ^4^ ^]^ and other solid cancers.^[^ ^5^ ^]^ An appropriate SLN tracer has been thought of as a key factor in successful SLN biopsy and further accurate LN staging. The ideal tracer is expected to possess the following favorable properties: i) they should rapidly reach SLNs, but cannot continue to drain to the second and third echelon LNs in a short time, so as to provide enough operation time window for surgeons;^[^ ^6^ ^]^ ii) the tracer had better show high sensitivity with a high spatial resolution to allow the surgeon to accurately locate SLNs with minimal invasion of surrounding tissues.

At present, the common methods of SLN identification include dye‐guided (e.g., methylene blue, MB),^[^ ^7^ ^]^ radioisotope‐guided (e.g., ^99m^Tc‐labeled sulfur colloid),^[^ ^8^ ^]^ and near‐infrared (NIR) fluorescence‐guided (e.g., indocyanine green, ICG) technique,^[^ ^6^, ^9^ ^]^ or their combinations.^[^ ^10^ ^]^ Although these approaches are used in clinics, all the previously mentioned methods suffer from some serious limitations. First, the injection time of ^99m^Tc‐labeled sulfur colloid needs 2–30 h before operation,^[^ ^11^ ^]^ which limits the surgery schedule. There is also the extra radiation risk of radioisotopes, which brings psychological burden to patients, surgeons, and nurses.^[^ ^12^ ^]^ Second, after injection of MB alone, the stained lymphatic vessels are thinner, so the anatomy of the LNs depends on a well clinical experienced surgeon to a great extent.^[^ ^13^ ^]^ Several studies mentioned that the detection rate of SLN by ICG method and dual‐tracer method could be improved,^[^ ^14^ ^]^ however, the main weakness is that the low molecular weight of MB and ICG causes the surgical time window too short. The short time window not only leads to unnecessary over‐dissection and node deletion^[^ ^15^ ^]^ but also fails to resist the re‐invasion of cancer cells.^[^ ^16^ ^]^ Overall, these studies highlight the need for new SLN tracking technology, which can provide a suitable surgical time window to meet clinical needs.

Surface‐enhanced Raman spectroscopy (SERS) plays a crucial and practical role in many fields, such as chemistry, materials, and medicine. The plasmonic nanoparticles generate a greatly enhanced electric field for the adsorbed Raman molecules at highly tunable wavelengths,^[^ ^17^ ^]^ resulting in high brightness and sensitivity of SERS nanotags. In addition, the characteristic fingerprint signals of Raman molecules could be distinguished from the adhering tissue and show better photostability than organic fluorescent dyes, which makes up for the deficiency of ICG in clinical applications. Last but not least, SERS nanotags have higher biological safety, which can avoid the inherent radioactive hazard of ^99m^TC‐labeled colloid. We have previously reported the core–shell structure plasmonic nanotags embedded with Raman reporters, named gap‐enhanced Raman tags (GERTs),^[^ ^18^ ^]^ whose SERS signals are highly reproducible due to the uniform distribution of electric fields in the nanogap and the protection of the outer shell for embedded reporters from environmental interferences.^[^ ^18^, ^19^ ^]^ With the unique advantageous features above, GERTs be deemed to a promising tracer that achieves intraoperatively pinpointing the SLNs in mice. However, for a real clinical transformative application, more following criteria should be fulfilled: i) enough surgical time window, SLN is not well demarcated because the traditional tracers diffusely drain into the high trapezoidal nodes; ii) a suitable animal model for simulation of the human lymphatic system; iii) preoperative positioning of SLN; iv) extensive evaluation of in vivo biocompatibility.

**Figure 1:**
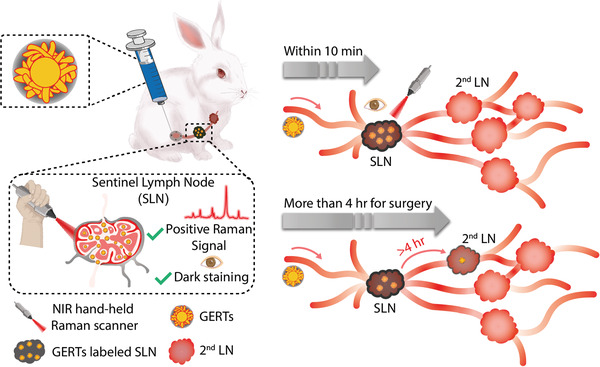
Schematic illustration of SLNs detection using Raman nanotags of GERTs. GERTs are injected subcutaneously before the surgery and migrate to SLNs after a gentle massage. SLNs can be in vivo identified from the unique Raman signal of GERTs within 10 min by a hand‐held Raman probe. GERTs only reach the 2^nd^ LNs after 4 h, which provides a suitable time window for surgery and avoids excessive resection of the LNs. Moreover, the ultrahigh brightness of GERTs allows the preoperative location of SLNs and enables the accurate removal of SLNs with minimal invasion.

In this work, we demonstrate GERTs as a novel tracer for SLN detection with assured safety and favorable properties. Rabbits were specifically selected as the animal model because their lymphatic drainage of the mammary gland is more similar to human beings,^[^ ^20^ ^]^ and all rabbit‐lymph‐node‐related surgeries were performed by an experienced breast surgeon. We have found that GERTs can quickly arrive within 10 min and stay in the SLN at least 4 h, thus providing an adequate operating window (**Figure** **1**). In addition, SLN labeled with GERTs can be identified by naked eyes due to its blue color of plasmonic colloids. A portable hand‐held Raman scanner was used to implement real‐time intraoperative detection of SLN and the confocal Raman system was further used for Raman imaging to verify the results on the portable scanner and also to investigate the dynamic migration of GERTs in SLN. Then, with the advantageous features including ultra‐strong Raman signal and unique fingerprint spectrum of GERTs, without slitting skin, we achieved preoperative SLN positioning and minimally invasive surgery. Finally, a comprehensive assessment of the biosafety of GERTs was conducted according to the requirements of the Food and Drug Administration (FDA) and the International Standard Organization (ISO), which can help promote the eventual clinical translation of this dual‐modal nanoprobe. This work shows that GERTs can realize sufficient surgical time window, preoperative positioning, and minimally invasive resection of SLN, which therefore holds promises as an excellent tracer.

## 2 Result and Discussion

### 2.1 Synthesis and Characterization of Raman Nanotags

**Figure 2:**
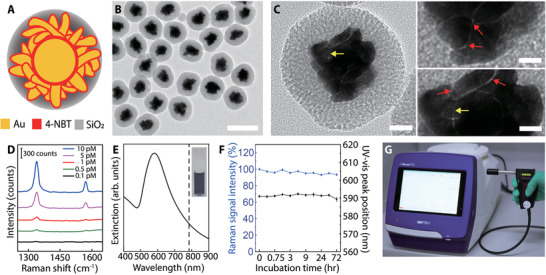
Characterization of GERTs. A) Schematic structural diagram of a GERT. Internal nanogaps in the core–shell structure and the rough petal‐like shell are decorated with Raman reporters of 4‐NBT and the particle is protected by a mesoporous silica layer. B) TEM image of a population of GERTs. The scale bar is 100 nm. C) Enlarged TEM images of a single GERT with multiple sub‐nanometer nanogaps (indicated by red and yellow arrows). The scale bars are 20 (left) and 5 nm (right). D) The detection limit of aqueous GERTs is 0.5 × 10^−12^ m (785 nm, 5.0 × 10^3^ W cm^−2^ power density, and 0.2 s acquisition time). E) Extinction spectrum of aqueous GERTs. The inset shows the corresponding photograph of aqueous GERTs (2.0 × 10^−9^ m). The dashed line indicates the laser excitation wavelength at 785 nm. F) Stability measurements of the GERTs in terms of extinction spectrum and Raman signal intensity (1340 cm^−1^). Data are means ± SD (*n* = 3). G) Photograph of a hand‐held Raman scanner.

Raman nanotags for LN localization prefer not only optical excitation in the NIR region to minimize the autofluorescence interference from tissues and the photodamage concern but also an optimal particle size (50–100 nm),^[^ ^21^ ^]^ high sensitivity, and stability in vivo. Core–shell‐structured GERTs (**Figure** **2**A) were synthesized according to our previous protocol.^[^ ^18^, ^22^ ^]^ Gold (Au) cores were prepared using a seed‐mediated process, modified with a Raman reporter of 4‐nitrobenzenethiol (4‐NBT), and further added into the mixed growth solution containing cetyltrimethylammonium chloride (CTAC), ascorbic acid, and chloroauric chloride (HAuCl_4_) to obtain the bare GERTs. Finally, the GERTs were coated with a silica layer.^[^ ^19^, ^23^ ^]^ The transmission electron microscopy (TEM) was utilized to characterize the core–shell morphology of GERTs. Figure 2B,C presents an overall appearance of GERTs which including a Au core, a sub‐nanometer gap embedded with reporter molecules, a petal‐like Au shell, and an outer mesoporous silica layer. The diameter of GERTs is about 80 nm, ideal for SLN imaging.^[^ ^19^, ^24^ ^]^ It is apparent from these figures that a few internal nanogaps between the Au core and shell (indicated by yellow arrows), as well as the external nanogaps between the petals (indicated by red arrows). The nanogaps could generate super‐strong electromagnetic fields when excited by 785 nm laser thus creating an enormous Raman signal enhancement with two featured bands at 1340 and 1575 cm^−1^. The aqueous GERTs show a limit of detection of 0.5 × 10^−12^ m based on the Raman peak at 1340 cm^−1^ (Figure 2D) using the hand‐held Raman spectrometer (Figure 2G). What stands out in Figure 2E are deep blue nanotags with a localized surface plasmon resonance (LSPR) peak at about 589 nm which is favorable for clinical applications since they can be well distinguished from normal tissue by the naked eyes, similar to MB tracer. Aqueous GERTs possess excellent stability at room temperature with a less than 10% fluctuation of Raman intensity (1340 cm^−1^) and a 2 nm shift of LSPR peak within 72 h (Figure 2F). The stability in the physiological environment such as phosphate‐buffered saline (PBS) and fetal bovine serum (FBS) were confirmed by further examinations of the Raman and extinction spectra over 72 h (Figure S1, Supporting Information). The above results have shown that these Raman nanotags are conductive to efficient LNs identification.

### 2.2 SLN Localization by GERTs using Portable Raman Scanner and Ex Vivo Raman Imaging

**Figure 3:**
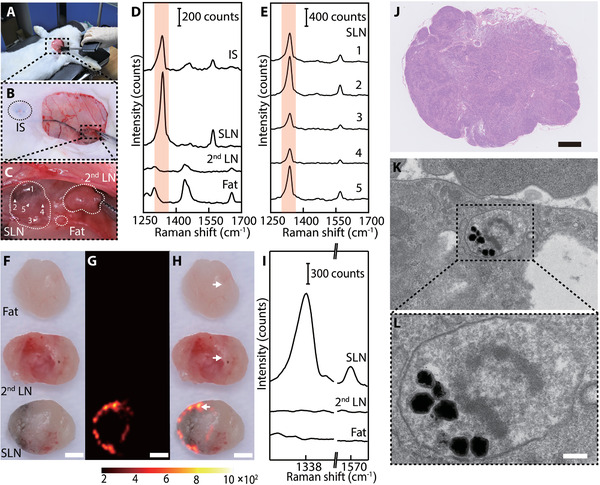
In vivo intraoperative LN tracing and ex vivo LN Raman imaging 10 min after injection of GERTs. A) Real‐time LNs tracing with a hand‐held Raman scanner (785 nm, 5.0 × 10^3^ W cm^−2^ power density, 0.2 s acquisition time). Raman signals were collected on the injection site (IS), LNs, and fat. B) Photograph of a rabbit after GERTs injection and skin removal. The black circle indicates the IS. C) Enlarged area (18 ×) indicated in panel (B). The white circles indicate the SLN, 2^nd^ LN, and the fat tissues. Raman spectra D) from different positions and E) five representative in situ scanning points (1: top, 2: left, 3: bottom, 4: right, 5: center) on the SLN. F–H) Photograph, Raman image, and the overlay of a dissected SLN, 2^nd^ LN, and adjacent fat tissue. Raman image was plotted using the Raman band at 1340 cm^−1^ (shaded area in (D)). I) Characteristic Raman signal of the position which marked by white arrows (in panel (H)) on the dissected SLN, 2^nd^ LN, and fat. J) H&E‐stained micrograph of a SLN section after GERTs injection. K,L) Bio‐TEM images of GERTs distributed in the organelle of lymphocyte. The scale bars are F–H) 1 mm, J) 500 µm, and L) 100 nm.

**Figure 4:**
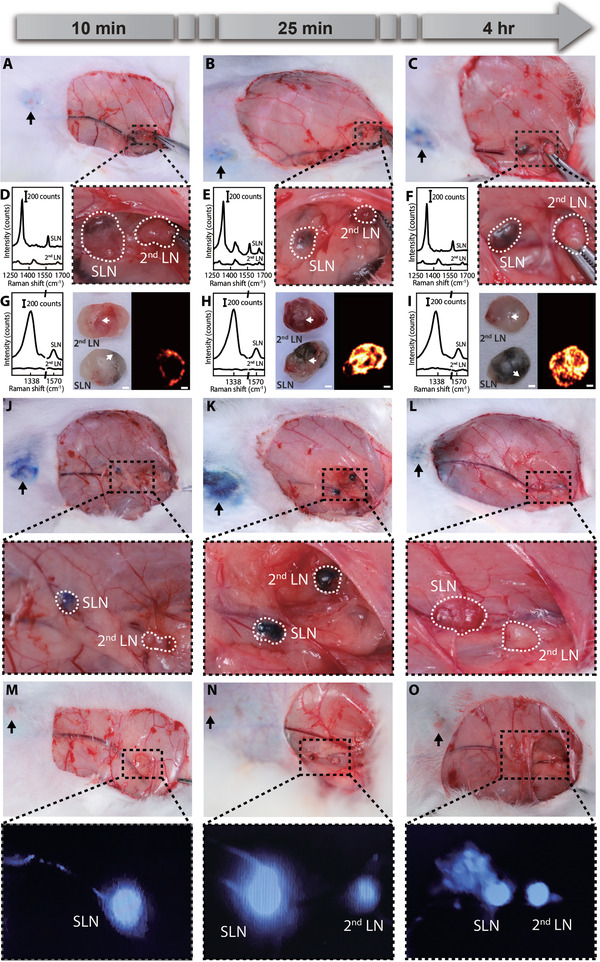
Comparison of in vivo LN tracing by GERTs, MB, and ICG. A–C) Photographs and enlarged images of SLN and 2^nd^ LN at different post‐injection time points of GERTs, and D–F) the corresponding in vivo Raman spectra. G–I) Ex vivo photographs, SERS images, and the corresponding Raman spectra from the positions indicated by white arrows on the dissected SLN and 2^nd^ LN (*n* = 3 for each time point). All Raman images are plotted using the characteristic Raman peak at 1340 cm ^−1^. The scale bars are G) 1000, H) 400, and I) 750 µm, respectively. J–L) Photograph and enlarged images of SLNs and 2^nd^ LNs after MB injection (*n* = 3 for each time point). M–O) Photographs and fluorescence images of SLNs and 2^nd^ LN after ICG injection (*n* = 3 for each time point). Black arrows are ISs of GERTs, MB, and ICG.

After characterization, we explored the feasibility of GERTs as an SLN tracer in the rabbit. For this purpose, the GERTs (150 µL, 2 × 10^−9^ m) were injected subcutaneously around the unilateral second nipple of rabbits (the one closest to the head is defined as the first nipple) and massage was implemented for 10 min as routine clinical practice before surgery (**Figure** **3**A). After a skin incision, the SLN showed slightly darker compared to the 2^nd^ LN and fat tissues (Figure 3B,C). Then the SLN was identified intraoperatively in situ by a hand‐held Raman scanner. It could be seen that the characteristic Raman band of GERTs at 1340 cm^−1^ was detected on the injection site (IS) and the SLN of the rabbit but not on the 2^nd^ LN or peripheral adipose tissue (see the shaded band in Figure 3D). Figure 3E shows the Raman spectra from five uniformly selected positions on the SLN and their different signal intensities enunciate the uneven distribution of GERTs inside the SLN. These findings were further corroborated by ex vivo Raman imaging of resected SLN, 2^nd^ LN, and fat using a confocal Raman system (Figure 3F–H). As it can be seen from the Raman image and bright‐field image, GERTs are mainly distributed around the margin of SLNs shortly after injection, consistent with our previous observation,^[^ ^19a^ ^]^ which can be understood by the fact that GERTs may reach the periphery of the SLNs through several afferent lymph vessels, and then eventually fill the entire SLN through the lymphatic sinuses due to the characteristic anatomical structure. In contrast, we did not detect GERT's signal from the 2^nd^ LN and adjacent fat tissue (Figure 3I), indicating the preferential accumulation of GERTs in the SLN. Examination of hematoxylin–eosin (H&E) staining further proves that the resected tissue is the LN tissue and there is no difference compared to SLN without GERTs by the observation of typical LN structures (Figure 3J). Bio‐TEM images ulteriorly manifested that some of the GERTs are phagocytosed by lymphocytes during the migration (**Figure** **4**K,L). At this stage, despite less amount of GERTs in the SLN, their Raman signals were strong enough for the hand‐held Raman system owing to the ultrahigh sensitivity. These results demonstrated that GERTs as an SLN tracer could locate the SLNs in ≈10 min efficiently and specifically during operation.

The ideal SLN imaging nanoprobes are expected to quickly arrive at and remain in the SLN for enough time to provide a sufficient period (at least 1 h) for the surgeon to distinguish SLN from 2^nd^ LNs.^[^ ^21a^ ^]^ To compare the performance of GERTs and MB (or ICG) to locate SLN and 2^nd^ LNs, the period of GERTs’ migration through the lymphatic vessels between the IS and the SLN was assessed using the hand‐held Raman spectrometer and additionally verified by Raman imaging via the confocal Raman system. By performing surgeries at different postinjection times, the dynamic migration process of nanotags was evaluated as shown in Figure 4A–C. The SLN could be labeled by GERTs with slight darkness within 10 min and the color turned black after 4 h. For 2^nd^ LNs, we not only failed to find the characteristic Raman signal of GERTs by the hand‐held Raman scanner in vivo (Figure 4D–F) but also did not observe obvious color change even at 4 h postinjection. Therefore, SLNs can be easily distinguished from 2^nd^ LNs in terms of Raman signals and the color within 4 h postinjection of GERTs. However, the characteristic Raman signal of GERTs could be collected on the SLN and the 2^nd^ LNs by the portable Raman detector in vivo 24 h after GERTs injection. In the meantime, the color of the 2^nd^ LNs marked by GERTs became darker. These phenomena indicate that GERTs migrate through the SLN and travel to the 2^nd^ LN after 24 h (Figure S2, Supporting Information). The GERTs‐labeled SLNs and 2^nd^ LNs were further resected for ex vivo Raman imaging at 10 min, 25 min, 4 h, and 24 h postinjection, and the results coincided well with the in vivo ones and demonstrated that GERTs could remain in the SLNs for at least 4 h (Figure 4G–I and Figures S2 and S3, Supporting Information), which is sufficient for the surgeon to perform intraoperative SLN resection. At 24 h postinjection, the distribution area of GERTs in SLN shrank and a clear signal appeared in the 2^nd^ LN. GERTs did not reach the 2^nd^ LN 24 h after the injection in our previous animal experiments in mice.^[^ ^19a^ ^]^ This can be possibly ascribed to the slow migration speed due to the narrower lymphatic vessels in mice compared to rabbits.

### 2.3 SLN Detection by MB or ICG

Considering the safety of radioactive tracers, MB and ICG, as common tracers of SLNs in clinical practice in China, are selected to perform comparative experiments on the rabbit model. Figure 4J–L shows the process of SLN tracking by MB. 10 min after injection, the SLN was roughly located based on experience, and then the skin and adipose tissue were removed. The SLNs with blue staining could be identified with the naked eye, while the color of the 2^nd^ LNs did not change significantly (Figure 4J). Due to a small molecular size, MB molecules pass readily through the SLN and do not remain in the SLN for a long time, so that 2^nd^ LN would be stained 25 min postinjection (Figure 4K), easily mistaking a nonsentinel node for SLN during the operation and leading to excessive LN dissection. After 4 h, the MB molecules were rapidly metabolized and excreted, thus blue staining disappeared in both SLNs and 2^nd^ LN (Figure 4L). This may result in a situation where an unskilled physician is unable to locate the SLN because of the fading of the dye. Figure 4M–O displays the process of SLN tracking by ICG. Transcutaneous detected fluorescent signals could quickly and directly trace LNs labeled by ICG. ICG reached SLN within 10 min (Figure 4M). However, it easily diffused through the SLN to the 2^nd^ LN, the same as MB dye, due to its low molecular weight. Consequently, the 2^nd^ LN was soon marked by ICG within 25 min (Figure 4N). The time window between the staining of the SLN and 2^nd^ LN is so short that misjudgment and excessive LN resection can occur during surgery. In addition, we also found that the fluorescent signal could be still detected in the SLN and 2^nd^ LN 4 h post‐injection. This is most likely explained by the fact that the ICG molecules penetrate into the surrounding tissues.^[^ ^25^ ^]^

### 2.4 GERTs‐Guided Positioning of SLNs for Minimally Invasive Surgery

**Figure 5:**
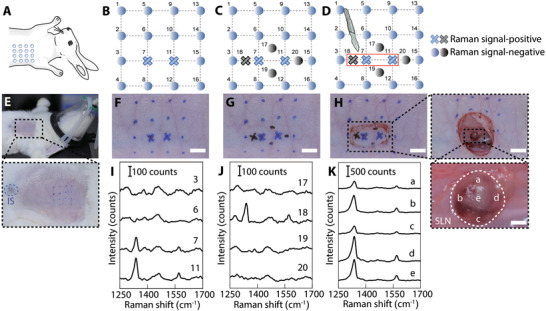
GERTs‐guided positioning of SLNs for minimally invasive surgery. A–D) The schematic of GERTs‐guided preoperative accurate location of SLN and minimally invasive surgery. E–H) Detection of Raman signals in the empirically predicted sentinel node region. Positive GERTs signal position marked as a cross (blue or black). I) Corresponding Raman spectra of skin‐covered negative (e.g., points 3 and 6) and positive points (e.g., point 7 and 11). J) Raman spectra of additional 4 points (point 17 to 20) around the positive points indicated in panels (C) and (G), measured through the skin. K) Raman spectra of five representative points on the SLN after removing the skin. The scale bars are 0.5 cm.

Preoperative detection can provide direct location prediction of SLNs, thereby further reducing the wound area and operation time. The hand‐held Raman prober again with a 785 nm laser to improve optical penetration in tissue. **Figure** **5** shows the process of GERTs‐guided identification of the SLN without slitting skin. GERTs (150 µL, 2 × 10^−9^ m) were injected subcutaneously at 4 points around the second nipple in an anesthetic rabbit. At 25 min postinjection, the surgeon first conducted a large‐area (1.5 cm × 1.5 cm, 16 points in total with an interval of 0.5 cm) scanning guided by a glass template before the skin removal (Figure 5A–E and Figure S5, Supporting Information). If positive Raman signals were detected, e.g., only at the position of 7 and 11 (Figure 5B,F–I and Figure S5B, Supporting Information). The point 7 and 11 were used as the centers and Raman scanning was performed again at points 17, 18, 19, and 20 to further accurately identify the area of the SLN (Figure 5C–G and Figure S5C, Supporting Information). The interval between points 18 and 7 is 0.25 cm which is the same as points 11 and 20, while points 17 and 19 are roughly 0.25 cm above or below the centers. For instance, if a positive Raman signal was found only at point 18 (negative at points 17, 19, and 20) (Figure 5J), the position of the SLN can be roughly identified, and consequently a skin cutting window of 1 cm × 0.5 cm (indicated by the red rectangle) can be determined (Figure 5D,H and Figure S5D, Supporting Information). The SLN can be finally identified after removing the skin with multiple positive Raman signals of GERTs on that area (Figure 5K). While a much larger surgical window (6 cm × 8 cm) is required in a typical MB‐guided lymphadenectomy (Figure S6, Supporting Information), which may increase unnecessary postoperative complications and require a longer time to recover.

### 2.5 Biological Safety Evaluation of Raman Nanotags

**Figure 6:**
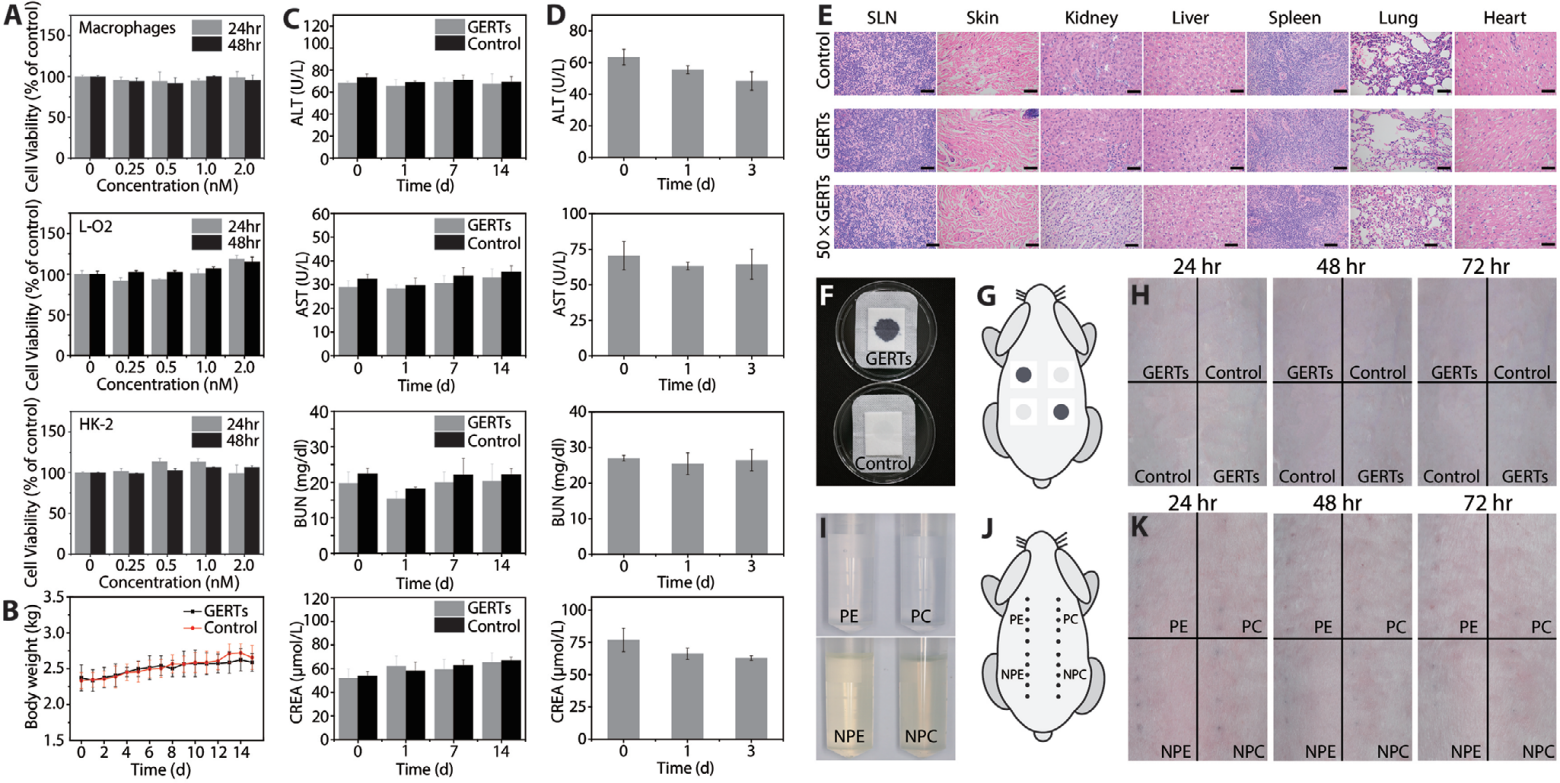
Biological safety evaluation. A) Cell viability of GERTs evaluated by co‐culture with macrophages, L‐O2, and HK‐2 cell lines for 24 and 48 h at different concentrations (*n* = 5 for each concentration). B) The effects of GERTs on rabbits’ body weight (*n* = 3 for each group). C) Influence of diagnostic dosage GERTs on blood biochemistry indices of the treated rabbits at 24 h, 7 d, and 14 d (*n* = 3 for each group). D) Blood biochemical indicators variation of rabbits with 50 times the diagnostic dosage of GERTs before and after injection at different time points (*n* = 3 for each group). E) Histological assessment of SLN, skin at the IS, liver, kidney, spleen, lung, and heart of the rabbits 14 days after injection. No significant pathological changes are found in treated rabbits. 50 × GERTs indicate the 50 times diagnostic dosage. The scale bars are 2 mm. Primary skin irritation evaluation (*n* = 3 for each group): F) GERTs and control solution on the sterile gauze dressing, G) indication of test sites of the rabbit, and H) photographs of evaluation sites at different post‐injection time points after removing the dressing. Intradermal reactivity test (*n* = 3 for each group): I) polar/nonpolar extraction solution of GERTs and its corresponding control solution, J) ISs on the rabbit, and K) the skin reaction at different post‐injection time points. Data are means ± SD (*n* = 3 or 5), *p* < 0.05.

Cytotoxicity, in vivo biosafety, the maximum tolerated dose test, the primary skin irritation test, and the intradermal reactivity test were examined to evaluate the biological safety of GERTs. Human normal liver cells (L‐O2), human kidney normal proximal tubule epithelial cells (HK‐2), and macrophages induced by human monocytic leukemia cells (THP‐1) were selected to assess the cytotoxicity of GERTs since nanoparticles generally have high‐level accumulation in the liver, are cleared and metabolized by the kidney, and are phagocytized by the reticular epithelial system.^[^ ^26^ ^]^ The media containing GERTs at concentrations from 0.25 × 10^−9^ to 2.0 × 10^−9^ m did not present any evidence of cell lysis or toxicity, as illustrated in **Figure** **6**A. Bodyweight, clinical signs, and biochemical index as the indicators of systemic toxicity were monitored on rabbits injected with saline or GERTs (1–14 days) according to ISO 10993‐11: 2017. During the observation, no abnormal clinical signs and animal deaths were found. Even with a slight increase in body weight, there was no significant difference between the control group and the GERTs group (Figure 6B). Measurements of biochemical indicators including serum alanine aminotransferase, aspartate aminotransferase, blood urea nitrogen, and serum creatinine showed that these indexes are at similar levels for all the rabbits treated with GERTs or saline (Figure 6C), indicating no detectable impact on the function of liver and kidney after injection of GERTs. No apparent pathological differences in liver, kidney, spleen, lung, heart, SLN, and skin were observed between the GERTs and the control group, which further confirms no appreciable change of systemic toxicity even after 14 days (Figure 6E). These results suggested that GERTs meet the requirement of acute and sub‐acute systemic toxicity tests.

In order to assess the potential safety issues caused by an overdose of GERTs and to consider animal welfare, the maximum tolerated dose test was adopted. The dose of GERTs administered (1.5 mL, 10 × 10^−9^ m) is 50 times higher than the actual diagnostic dose (150 µL, 2.0 × 10^−9^ m). Biochemical indicators and histological assessment of the main organs of treated rabbits show no significant abnormal signs including morphology or inflammatory indications (Figure 6D,E). These preliminary tests show that GERTs are less likely to produce toxicity at the diagnostic dose.

During the primary skin irritation test, skin reactions at various time points were recorded (Figure 6F,H and Figure S7, Supporting Information), no animal deaths and aberrant clinical signs caused by the GERTs (150 µL, 2 × 10^−9^ m) are found. Skin responses at 24, 48, and 72 h postinjection showed that the control and test groups did not cause erythema, edema, and any other skin reactions. Skin abnormalities such as erythema or edema were not observed at 24, 48, and 72 h postinjection between the control and GERTs group. Thus, the sensitization scores after 24, 48, and 72 h challenges are all determined to be 0 according to ISO 10993‐10: 2010.

In the intradermal reactivity evaluation, mild erythema was observed on two rabbits due to the hair removal process, so saline or cottonseed oil extract of GERTs (150 µL, 2 × 10^−9^ m) were injected after the erythema disappeared. No distinct clinical reactions and animal deaths were found throughout the test; the ISs and local response observation 0, 24, 48, and 72 h after injection suggest that erythema, edema, eschar, and skin response were not detected (Figure 6I,K). Therefore, the cumulative irritation index (CII) values calculated for different extracts of the test and control group were below 1.0, indicating that the GERTs exhibit negligible intradermal reactivity according to the criteria of the ISO guidelines. Skin reactions at different time points are also supplemented in Figure S8 in the Supporting Information.

In this work, we chose the rabbit model because it is more suitable than mouse for simulating human being in the following aspects. First, the lymphatic system of rabbits is more similar to that of humans in terms of the lymphatic territories, the diameter of the lymphatic vessels and the lymphatic drainage of the mammary gland. The rabbit has eight lymphatic territories (Figure S9, Supporting Information) and except the lateral sacral (labeled as 8), all the others are also observed in human, but usually do not normally exist in mice or rats.^[^ ^27^ ^]^ Several studies have also reported that the diameter of lymphatic vessels in rabbits is 170–1300 µm,^[^ ^28^ ^]^ much closer to that in humans (200–2000 µm)^[^ ^29^ ^]^ compared to mice (65–155 µm).^[^ ^30^ ^]^ Additionally, rabbit bears a resemblance to human being in terms of the lymphatic drainage of the mammary gland.^[^ ^31^ ^]^ Axillary accessory dorsal and ventral LNs in rabbits are comparable with pectoral and apical nodes of axillary LNs in humans. Similar to humans, rabbits also possess superficial and deep lymphatic drainage systems in the upper extremities,^[^ ^32^ ^]^ whereas this dual pathway is not well constructed in mice and rats. Next, it is difficult to investigate the suitable diagnostic parameters (e.g., dosage, concentration, and retention duration of tracers) for human beings in mice model due to the small volume of rodents.^[^ ^20^, ^33^ ^]^ It has also been found that the healthy rabbit can be an effective animal model for the preclinical evaluation of SLN tracers instead of building up a tumor model.^[^ ^34^ ^]^ On account of the above reasons, the rabbits were chosen for SLN identification experiments.

**Figure 7:**
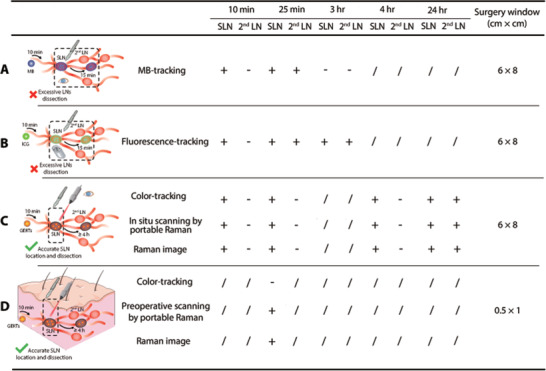
Comparison of various strategies for SLN identification. A) MB tracking by naked eyes: SLN and 2^nd^ LN can be blue‐stained in 10 and 25 min, respectively; they are both cleaned after 3 h. B) ICG tracking by the portable fluorescent detector: SLN and 2^nd^ LN can be labeled in 10 and 25 min, respectively; they maintained labeled within 3 h. C) GERTs guidance by naked eyes, the portable Raman detector, and Raman imaging: SLN can be labeled in 10 min and show unchanged within 24 h; 2^nd^ LN is unlabeled in 4 h and show positive after 24 h. All intraoperative SLNs locations typically lead to a surgery size window of about 6 cm × 8 cm. D) Preoperative GERTs guidance by the portable Raman detector: SLN can be detected at 25 min postinjection. The preoperative SLN location leads to a window of about 0.5 cm × 1 cm. +/− represents the positive/negative signal of MB, ICG, or GERTs, and / indicates that no tests were performed at that time.

Compared with traditional tracers (**Figure** **7**), we show that GERTs hold great promises as a tracer to achieve precise intraoperative positioning of SLN using a portable Raman spectrometer and to provide enough time for surgery. In the clinic, even if the tracers with a small molecular size like MB and ICG can quickly circulate in the lymphatic vessels to achieve rapid positioning, they easily drain into the 2^nd^ LN soon (Figure 7A,B). The LNs beyond the sentinel area are marked in such a short time window (less than 25 min) that it is difficult for even experienced surgeons to distinguish between SLN and non‐SLN, so they must remove all lymph nodes labeled by the tracer.^[^ ^35^ ^]^ As an ideal SLN tracer, GERTs could remain in SLN for at least 4 h without draining into the 2^nd^ LN (Figure 7C), providing the surgeon with sufficient time to distinguish SLN from 2^nd^ LN and adipose tissue, thereby effectively avoiding excessive lymph node dissection. It can be found that Raman nanotags can remain in the SLN of mouse for over 24 h, which is much longer than that in the rabbit model.^[^ ^19a^ ^]^ The retention time of two animal models in SLNs is different, which can be explained by the following facts. First, there are obvious differences in the lymphatic system between the mouse and rabbit as we pointed out above in terms of the lymphatic vessel diameter and the lymphatic drainage of breast. The lymphatic vessels in mouse are narrower than that in rabbit,^[^ ^28^, ^30^ ^]^ and the SLN of mouse is relatively far from the 2^nd^ LN, which is beyond the scope of regional LNs (Figure 4 and Figure S10, Supporting Information). Second, 0.5 wt% polyvinylpyrrolidone (PVP) is additionally added as a stabilizer to improve the dispersion of the nanotags and to avoid the agglomeration in this work. This may also improve the migration of nanotags in the lymphatic system in vivo.^[^ ^36^ ^]^ Third, the SERS performance of ultrabright petal‐like GERTs (P‐GERTs) used in this work is improved, which makes it possible to be detected even if only a small amount of nanotags reach the 2^nd^ LN.^[^ ^19b^ ^]^ In addition, precise SLN dissection saves more LNs for patients to resist the tumor re‐invading, improve the outcomes, and protect poor physical situation.^[^ ^16^, ^37^ ^]^ Furthermore, GERTs makes it possible to implement rapid in situ positioning of SLN as the traditional tracers. They reached SLN within 10 min, and characteristic signals could be detected by a portable Raman spectrometer owing to high sensitivity. A commercial portable Raman spectrometer can fulfill GERTs‐guided intraoperative SLN tracing, thereby making up for the trouble that hospitals with poor medical resources cannot afford expensive imaging equipment based on radionuclide and fluorescence. Also, the SLNs marked by GERTs appear black, so they can be determined directly by the naked eye, which is operable and practical in surgery. These above‐mentioned superiorities are that, on the one hand, disadvantages (e.g., long‐time staining of ISs and autofluorescent) caused by MB and ICG are minimized (Figure S11, Supporting Information), and on the other hand, trouble caused by multiple injections of tracers is also reduced.

Location of SLN before skin incision could greatly reduce the surgical trauma and further improve prognosis, which is a vital clinical preference. Even if SLN located near the skin can be detected percutaneously by radiotracking technology, it may introduce unnecessary radiation exposure.^[^ ^12^ ^]^ Besides, the use of radiotracer forces hospitals to deal with a series of unnecessary logistical issues such as isotope disposal and personnel training, as well as the requirements to set up a special nuclear medicine department. It should be noted that the excitation of the ICG could be also detected transcutaneously real‐time using a high‐sensitive camera, but the shortcomings of rapid drainage to 2^nd^ LN and susceptibility to interference from autofluorescence making the location of the SLN still needs to be determined based on the doctor's experience. GERTs‐guided preoperative SLN positioning paves the way for minimally invasive surgery (Figure 7D). The realization of the above ideas is based on the following advantageous points: i) ultrabright GERT probes, which can be detected even at very low concentrations without slitting skin; ii) the stability and nonaggregation of GERTs in various physiological conditions, ensuring their successful drainage from the IS to the SLN. Employment of GERTs not only greatly reduces surgical wounds by 80 times but also shortens the operation time. Moreover, GERTs combined with other Raman techniques, such as spatially offset Raman spectroscopy^[^ ^38^ ^]^ and endoscopic Raman,^[^ ^39^ ^]^ providing solutions to the tumor (e.g., gastrointestinal cancer, breast cancer, glioblastoma multiforme)^[^ ^40^ ^]^ surgeries where the SLN biopsy is needed in deep tissue.

The above extensive biological safety evaluations have demonstrated no noticeable adverse effect of GERTs. It is rational to consider that the biological toxicity of GERTs is negligible by further taking into account three following factors: i) the removal of SLN can reduce the total amount of nanotags left in the body to a certain extent; ii) no obvious in vivo biosafety issues have been found in recent comprehensive evaluations of Au‐SiO_2_ composite nanoparticles, which are similar in size and composition to GERTs;^[^ ^41^ ^]^ iii) in this work, GERTs are applied in a scenario of subcutaneous injection, which is safer by considering the capability to spread to nontarget tissues compared with intravenous injection; iv) more and more other Au‐SiO_2_ nanoparticles for therapeutic and imaging purposes have been tested in clinical trials,^[^ ^42^ ^]^ which has greatly promoted the clinical translation of GERTs.

## 3 Conclusion

In summary, we demonstrate that GERTs as a more suitable tracer enable rapid and accurate intraoperative positioning of SLNs as quickly as traditional tracers (e.g., MB and ICG) in vivo with the use of a portable Raman scanner. GERTs are essentially useful for dual‐mode (color/Raman signal) SLN identification and can realize the integration of surgeon judgment and instrument guidance. The retention time (>4 h) of GERTs in SLNs is suitable and long enough to avoid the misjudgment of non‐SLNs caused by too fast migration of the tracers, thus effectively circumventing excessive lymphadenectomy. In vitro Raman imaging of SLN, 2^nd^ LNs, and fat tissue was further performed on a Raman confocal system to investigate the dynamic migration behaviors of GERTs in the lymphatic system, confirming a surgical window of more than 4 h. The extensive biosafety examinations including cytotoxicity, in vivo biosafety, the maximum tolerated dose test, the primary skin irritation test, and the intradermal reactivity test has shown no significant or systematic toxicity of GERTs. Although additional studies maybe are required to investigate the long‐term effects of GERTs, the superiorities including portability, cost‐effectiveness, and biosafety are greatly favorable for the requirements of clinical translation. With the continuous development of portable Raman spectrometers combined with the improvement of nanoparticles, it is conceivable that the clinical application will not only be limited to SLN tracing but also can be broadened to biosensing and bioimaging fields, thereby realizing a clinical multidisciplinary diagnosis platform.

## 4 Experimental Section

This study was designed to develop an efficient and minimally invasive method for intraoperative real‐time detection of SLNs using the SERS technique. To better simulate human breast lymph node drainage, the New Zealand rabbit was selected, whose lymphatic drainage of the breast is more similar to the human. The reflux of breast lymph nodes in rabbits was mainly through the axillary accessory dorsal and ventral LNs. Axillary accessory dorsal LNs of the rabbit were superficial, while axillary accessory ventral LNs were profound and very close to the axillary vein. According to anatomical location and direction of lymphatic drainage, axillary accessory dorsal LNs were defined as SLN and axillary accessory ventral LNs as 2^nd^ LN in the rabbit model in this study. After the synthesis and characterization of GERTs, it was begun to evaluate the possibility of GERTs as the SLN tracer and comparing them with the standard clinical tracers such as MB or ICG. The feasibility of GERTs‐guided identification of SLNs was assessed in situ using a portable Raman spectrometer to mimic the intraoperative operation. Especially, the time for the tracers draining from the SLN to the 2^nd^ LN was investigated. This important time window directly determined the possibility of clinical accurate SLN removal without causing excessive LNs dissection. Raman imaging with a confocal microscopic Raman spectrometer was applied to confirm the reliability of the results obtained with the portable Raman spectrometer and to investigate the dynamic migration of GERTs in the lymphatic system over time. The detection of GERTs signals was further implemented without a skin incision, which could locate SLN before surgery and realize minimally invasive biopsy of SLNs. In addition, the cytotoxicity and biological safety evaluation of GERTs according to the International Organization for Standardization (ISO) was also evaluated, laying a foundation for future clinical translation. All animal experiments were performed in compliance with the Animal Care and Use Committee of the School of Medicine, Shanghai Jiao Tong University.

### Chemicals and Reagents

Chloroauric chloride (HAuCl_4_⋅H_2_O), ascorbic acid (> 99%), tetraethyl orthosilicate (TEOS), and sodium hydroxide (NaOH) were acquired from Sinopharm Chemical Reagent Co. Ltd. Cetyltrimethylammonium chloride (CTAC) and sodium borohydride (NaBH_4_) were purchased from J&K Chemical Ltd. (Shanghai, China). 4‐Nitrobenzenethiol (4‐NBT) was received from Sigma‐Aldrich (Shanghai, China). Indocyanine green (ICG) was from Dandong Yichuang Pharmaceutical Co. Ltd. and methylene blue (MB) was purchased from Jumpcan Pharmaceutical Group Co. Ltd. PVP (K30, Mw = 44 000–54 000) was purchased from Aladdin (Shanghai, China). Nanopure water (18.2 MΩ) was used for all experiments. All materials were used as received without any further purification.

### Synthesis and Characterization of Raman Nanotags

GERTs were synthesized as described in previous protocols.^[^ ^23^ ^]^ First, a seed‐mediated method was used to prepare uniform‐sized Au cores (0.48 × 10^−9^ m). Before adsorbing 4‐NBT molecules on the Au core, the concentration of CTAC was decreased from 100 × 10^−3^ to 20 × 10^−3^ m by centrifugation to reduce its influence on the adsorption process of 4‐NBT. Then 100 µL 4‐NBT ethanol solution (10 × 10^−3^ m) was added dropwise to the 2 mL Au cores under ultra‐sonication. After incubation for 5 min, the Au cores were washed by centrifugation. 960 µL 4‐NBT absorbed Au cores solution (0.94 × 10^−9^ m) as seeds and 480 µL ascorbic acid (40 × 10^−3^ m) were sequentially transferred to a mixture of 16 mL CTAC (50 × 10^−3^ m) and 800 µL HAuCl_4_ (4.86 × 10^−3^ m) rapidly under vigorous sonication to form the core–shell structure by reduction of Au^+^. Finally, a mesoporous silica layer was coated around the Au shell as follows: 40 mL of the above obtained GERTs was washed with water and centrifuged three times, and then dispersed in CTAC solution (5 mL, 5 × 10^−3^ m). After adjustment of pH to 10.4 by NaOH solution (0.1 m), 50 µL of 5% TEOS in methanol was added to the solution with stirring in the water bath (35 °C). This addition was repeated three times at 30 min intervals, and then the reaction continued for 17 h. Finally, the resultant product was washed twice with ethanol and NH_4_NO_3_ to obtain mesoporous silica‐coated GERTs.

TEM images were collected on a JEM‐2100F TEM (JEOL, Tokyo, Japan) operated at 200 kV. UV‐Vis spectra were obtained from a UV1900 UV‐Vis spectrophotometer (Aucybest, China). The investigation of the detection limits and stability of aqueous Raman nanotags and the intraoperative/preoperative SLN detection were all carried out on a portable hand‐held Raman spectrometer (i‐Raman Plus, B&W Tek) with a spectral resolution of 4.5 cm^−1^ and a 785 nm NIR laser (5.0 × 10^3^ W cm^−2^ power density and 0.2 s acquisition time). The Raman scanner was connected to a laptop installed with the BWSpec software.

### MB, ICG, and GERTs‐Guided Intraoperative SLN Positioning

First, female New Zealand rabbits (*n* = 9 for each tracer, total of 3 time points, 3 rabbits at each time point, 2.5−3.5 kg, 3 months, clean degree, Shanghai Jiagan Biological Technology Co., Ltd.) were anesthetized with 2.5% isoflurane. Female rabbits have four pairs of nipples, the one near the head is defined as the first nipple, and so on. Then, 150 µL MB (10 mg mL^−1^) was administered into interstitial breast tissue around the second nipple at 4 points by subcutaneous injection and subsequent massage for 10 min as common clinical practice. After that, the surgeon dissected rabbits at 3 different time points (3 parallel experiments at each time point) to investigate the distribution of MB in the lymphatic system over time. Photographs of MB‐labeled LNs were taken by a Canon camera (EOS 5DS). Then the dissected LNs were fixed overnight in 4 °C paraformaldehyde (4%), then embedded in paraffin, and stained with H&E for further histological analysis according to previous method.^[^ ^19a^ ^]^ The process of ICG for tracking SLNs was followed similarly with an injection dose of 150 µL (0.25 mg mL^−1^). Fluorescence images were collected by a portable fluorescence imaging system (MDM‐I, Ming De Medical Diagnosis).

Following a similar process as MB‐guided SLN tracking, anesthetized rabbits (*n* = 12, total of 4 time points, 3 rabbits at each time point) were injected subcutaneously with GERTs in 0.5 wt% PVP‐saline (150 µL, 2 × 10^−9^ m, 0.5 wt% PVP‐saline) at 4 points around the second nipple. After massaging for 10 min, the surgeon dissected the skin near the axilla of the rabbit forelimb. Photographs of LNs stained with GERTs and the Raman spectra of SLN, 2^nd^ LN, and adjacent fat were collected in situ by using the hand‐held Raman scanner with a laser power density of 5 × 10^3^ W cm^−2^ and an acquisition time of 0.2 s, which were optimized to prevent tissue damage. An attachment in front of the probe was applied to maintain the suitable focus of the laser on the surface of the SLNs with a distance of about 5.8 mm. Raman scanning was performed typically at 5 positions to confirm each SLN.

### Raman Imaging of Ex Vivo LNs and Fat

After different drainage periods, GERT‐labeled LNs (including SLNs and 2^nd^ LNs) were confirmed by measuring the characteristic Raman signal with a hand‐held Raman scanner and the subsequent excision of the entire LNs was carried out along the external contours. The adipose tissue near the SLNs was also dissected for comparison. 2^nd^ LNs that were not marked by GERTs were located and removed based on the experience of the surgeon on the anatomical location. All the Raman images of resected tissues were acquired by a confocal Raman system (LabRAM XploRA INV, Horiba) with a STAGE mapping mode, using a 785 nm laser (3 × 10^5^ W cm^−2^ power density), 1 s acquisition time per spectrum, a 10 × objective, and a step size of 100 µm. During the STAGE mapping mode, the Raman spectrum of each point of a sample was recorded by the movement of the stage to cover the entire tissue. The characteristic band at 1340 cm ^‐1^ of GERTs was used to generate all Raman images using the LabSpec 6 software (Horiba).

### GERTs‐Guided Positioning of SLNs for Minimally Invasive Surgery

Anesthetized female rabbits (*n* = 3, 2.5−3.5 kg, 3 months) were subcutaneously injected with GERTs (150 µL, 2 × 10^−9^ m) around the second nipple in 4 points. 25 min postinjection, the surgeon performed large‐area scans to roughly locate SLN after hair removal by using a hand‐held Raman scanner. 16 points were selected uniformly in the scanning area (1.5 × 1.5 cm^2^, 0.5 cm interval between each point), which was based on the surgeon's clinical experience for Raman signal detection. When a positive Raman signal was detected at a certain point/area, the Raman scanning was performed again under the same signal acquisition parameters in the direction of 3, 6, 9, and 12 o'clock with an interval of 5 mm to narrow the area of SLN. After accurately locating the position of SLN based on the Raman scanning, the surgeon performed minimally invasive surgery to remove SLNs for subsequent biopsy.

### Cytotoxicity

Cell Counting Kit‐8 (CCK‐8) (Dojindo, Japan) assay was applied to evaluate the potential of cytotoxicity. For cell viability study of GERTs, human normal liver (L‐O2) cell (5.0 × 10^3^ cells per well), human kidney normal proximal tubular epithelial (HK‐2) (5.0 × 10^3^ cells per well), and macrophages cells induced by human monocytic leukemia cell (THP‐1) (1.0 × 10^5^ cells per well) were cultured in 96‐well plates for 24 h in Dulbecco's modified Eagle medium (DMEM)/high glucose, DMEM/F‐12 (Ham) medium, and Roswell Park Memorial Institute (RPMI) 1640 medium, respectively, supplemented with 10% FBS, 100 U mL^−1^ penicillin G, and 100 U mL^−1^ streptomycin at 37 °C in a humidified atmosphere containing 5% CO_2_. After the adherence of cells, the culture medium was refreshed and the cells were co‐cultured with GERTs at various concentrations (0 × 10^−9^, 0.25 × 10^−9^, 0.5 × 10^−9^, 1.0 × 10^−9^, and 2.0 × 10^−9^ m, *n* = 5 for each concentration) for an extra 24 or 48 h. At least five parallel tests were set for each concentration of GERTs and the other three wells with only medium and GERTs (without cells) were applied to remove the influence of optical absorption from GERTs at 450 nm. After rinse three times with PBS, 100 µL fresh culture medium containing 10 µL CCK‐8 solution was added into each well. After incubation for 2.5 h, the optical density (OD) was measured with a Microplate Reader (Bio‐Rad, USA) at 450 nm. Values in the text were expressed as the means ± standard deviation (SD), and *p* < 0.05 was considered statistically significant.

### Acute and Subacute Systemic Toxicity Test

Six female rabbits (2.5−3.5 kg, 3 months, *n* = 3 for each group) were randomly divided into the control group and GERTs group. Before dosing, all rabbits were weighted according to ISO 10993‐11:2017. Three rabbits in the experiment group were injected subcutaneously with GERTs in 0.5 wt% PVP‐saline (150 µL, 2 × 10^−9^ m) around the second nipple in 4 points, while the other three rabbits in the control group were injected with an equal volume of saline in the same injection method. If none of the animals treated with GERTs showed significantly higher biological reactivity than those in the control group treated with saline within 24 h after the injection, the Raman nanotags met the requirements of the acute systemic toxicity test. Similarly, if 14 days after the injection, compared with the control group, the experimental group did not show any obvious abnormalities, then it met the requirements of the subacute toxicity test. The rabbits were observed and weighed every day for 14 days. If behavior such as shortness of breath, cramps, or diarrhea occurred, or if a bodyweight loss of more than 10% occurred in the GERTs group, the sample did not meet the requirements of the systemic toxicity test. The blood was collected before injection and at 24 h, 7 d, and 14 d postinjection from the vein at the ear edge. About 500 µL whole blood was collected once per rabbit and the plasma in the whole blood was obtained by centrifugation for biochemical analysis to evaluate the function of the liver and kidney. At the last time point, the rabbits were euthanized after collecting blood and major organs including heart, liver, spleen, lung, kidney, SLN, and skin at the injection site were harvested to evaluate the morphologic changes by histopathologic analysis. The slices were examined by optical microscopy (Olympus Medical Corporation, Tokyo, Japan). All institutional and national guidelines for the care and use of laboratory animals were followed.

### Primary Skin Irritation Evaluation

Three female rabbits (2.5−3.5 kg, 3 months) were involved in the primary skin irritation assessment of the nanotags according to ISO 10993‐10: 2010. 24 h before evaluation, the rabbit's back fur was clipped and divided into four regions with the same area (40 mm × 40 mm). Sterile dressing was infiltrated by GERTs solution (0.5 mL 0.5 wt% PVP‐saline, 2 × 10^−9^ m) according to the manufacturer's directions and immediately applied to two sites; the other two sites were used as a control (0.5 mL saline). After wrapping the treatment sites and the control sites on the back with a nonocclusive bandage, the animals were returned to the cages. 4 h later, the rabbits were then examined for the allergic reaction at the application site after removing the bandage and nanotags. After 24, 48, and 72 h, the examinations were repeated for the area with nanotags and saline. The reaction, defined as erythema or edema, was evaluated according to the score of the skin reactions reported in ISO 10993‐10: 2010.

### Intradermal Reactivity Test

Three female rabbits (2.5−3.5 kg, 3 months) were used to assess the reactivity of the Raman nanotags within dermal after intradermal injection of 0.2 mL GERTs polar or nonpolar extracts at five sites on one side of the spine of each rabbit according to ISO 10993‐10: 2010. GERTs (150 µL, 2 × 10^−9^ m) were extracted with saline or cottonseed oil as the extractant to obtain the polar or nonpolar extract. Similarly, 0.2 mL extraction vehicles without test articles as negative control were injected on five sites of the contralateral side of each rabbit. After 24, 48, and 72 h, the ISs were examined for the erythema and edema, and then scores were obtained according to their severity for each test solution. After the calculation of each animal, the total grading was divided by 3 to obtain the mean score for each solution including the negative control. The requirements of the test were met if the difference between the test solution and the control solution mean score was 1.0 or less. If the difference between the mean score of the test solution and the control solution was 1.0 or less, the intradermal reactivity test requirements were met. All institutional and national guidelines concerning the care and use of laboratory animals were followed. The above‐mentioned in vivo tests were performed according to ISO 10993 series.

### Maximum Tolerated Dose Test

The median lethal dose was not used to study the biosafety of GERTs considering animal welfare. Instead, 50 times the diagnostic dose of nanotags was adopted, i.e., 1.5 mL, 10.0 × 10^−9^ m, for this evaluation with reference to the SLN tracer Magtrace approved by the FDA.^[^ ^43^ ^]^ Three female rabbits (2.5−3.5 kg, 3 months) were injected subcutaneously with GERTs in 0.5 wt% PVP‐saline (1.5 mL, 10.0 × 10^−9^ m) around the second nipple in 4 points. For each rabbit, their physical appearance (fur, eyes), behavior (posture, gait, breathing pattern), and interactions toward other animals were observed and recorded daily. Also, 500 µL whole blood was collected from each rabbit via the vein at the ear edge 1 day before Raman nanotags administration to determine the baseline blood chemistry and at 24 and 72 h postinjection to evaluate the liver and kidney function. At the last time point, the rabbits were euthanized after collecting blood and major organs were harvested for histopathology analysis.

### Statistical Analysis

In this work, only the UV‐vis spectra were normalized. All Raman spectra collected from the confocal system or the portable spectrometer and the other data not specifically noted were raw data without any pre‐processing. To evaluate the cytotoxicity of GERTs on cell lines, each GERTs’ concentration was set in quintuplicate while the control group was in triplicate. In the animal experiments, three rabbits were used in each group including the experimental and the control group. Each plasma sample was tested three times. The mean and standard deviation of biochemical index were calculated to verify whether there is liver cell damage or renal dysfunction. Detailed information on the sample size was also described in the figure legends. All values in figures were presented as means ± SD unless otherwise noted in the text and figure legends. Statistical significance was calculated by Excel software based on the Student's *t*‐test and the level of significance was set at *p* < 0.05.

## Conflict of Interest

The authors declare no conflict of interest.

## Author Contributions

B.D. and Y.H.W. contributed equally to this work. B.D. designed and synthesized the nanoparticles, performed LNs resections and Raman detection/imaging, analyzed the data, and wrote the manuscript. Y.H.W. performed LNs resections, analyzed the data, and wrote the manuscript. Y.W. performed animal experiments and took photographs. J.Y., J.L. and W.Y. supervised and coordinated the study, designed the experiments, analyzed data, and revised the manuscript. The manuscript was critically evaluated and approved by all co‐authors.

## Supporting information

Supporting Information

## Data Availability

Data are available on request from the authors.
