# Supplementary material for: Raman Nanotags‐Guided Intraoperative Sentinel Lymph Nodes Precise Location with Minimal Invasion
Source: Adv Sci (Weinh). 2021 Nov 5;9(2):2102405. doi: 10.1002/advs.202102405 (PMC8805599; doi:10.1002/advs.202102405)
Supplement: Supplementary file 1 — Supporting Information [file ADVS-9-2102405-s001.pdf]

ADVANCED SCIENCE
Open Access

Supporting Information

for *Adv. Sci.*, DOI: 10.1002/advs.202102405

Raman nanotags-guided intraoperative sentinel lymph nodes precise location with minimal invasion

Binge Deng, Yaohui Wang, Yifan Wu, Wenjin Yin, Jinsong Lu*, Jian Ye**

Supporting Information

Raman nanotags-guided intraoperative sentinel lymph nodes precise location with minimal invasion

Binge Deng, Yaohui Wang, Yifan Wu, Wenjin Yin, Jinsong Lu*, Jian Ye**

Dr. B Deng, Prof. J Ye
State Key Laboratory of Oncogenes and Related Genes, School of Biomedical Engineering
Shanghai Jiao Tong University
Shanghai 200030, P. R. China
E-mail: yejian78@sjtu.edu.cn

Dr. Y Wang, Dr. Y Wu, Dr. W Yin, Prof. J Lu
Department of Breast Surgery, Renji Hospital, School of Medicine
Shanghai Jiao Tong University
Shanghai 200127, P. R. China
E-mail: yinwenjin@renji.com, lujingsong@renji.com

Prof. J Ye
Shanghai Key Laboratory of Gynecologic Oncology, Renji Hospital, School of Medicine
Shanghai Jiao Tong University
Shanghai 200127, P. R. China

Prof. J Ye
Institute of Medical Robotics
Shanghai Jiao Tong University
Shanghai 200240, P. R. China

The PDF file includes:

Figure S1. Stability measurements of UV-vis extinction spectra and Raman intensity of GERTs incubated with (A) 0.5% PVP, (B) 0.5% PVP-Saline and (C) 0.5% PVP-10%FBS. The extinction spectrum of GERTs in 10% FBS is not recorded since the absorption of FBS overlaps with the resonance peak of GERTs.

Figure S2. Evolution of in vivo LN tracing and ex vivo LN SERS imaging at post-injection 24 h of GERTs. (A) Photographs and enlarged images of SLN and 2nd LN at different post-injection time points of GERTs, and (B) the corresponding in vivo SERS spectra. (C) *Ex vivo* photographs, SERS images and the corresponding SERS spectra from the positions indicated

on the dissected SLN (arrow a) and 2nd LN (arrow b). All Raman images are plotted using the Raman band at 1338 cm^{-1}. The scale bars are 400 μm.

Figure S3. SERS and merge image of a dissected SLN and 2nd LN at different time points after GERTs injection. The scale bars are (A) 400 μm, (B) 750 μm and (C) 400 μm, respectively.

Figure S4. Total Raman spectra of 16 detection points by portable Raman scanner during SLN subcutaneous positioning. The acquisition parameter is 785 nm laser excitation, 400mW laser power density and 0.2s acquisition time. The scale bar is 5 mm.

Figure S5. GERTs achieve accurate preoperative positioning of SLN. (A to D) The marks on the skin are drawn with the same size on the glass, which used to confirm the SLN position after dissecting the skin. (E) Placed the glass slide over the skin and match the dots on the glass with the dots on the skin one by one while skin dissected. The point 7 on the slide completely coincides with the position of SLN in vivo. (F) Marks on the glass slide. (G) Light image and merge image of dissected SLN by a Raman confocal system. All Raman images are plotted using the Raman band at 1338 cm^{-1}. The scale bar are (B, C, E) 5 mm and (G) 200 μm.

Figure S6. Comparison of surgical window size for SLN resection after GERTs (A) and MB (B) labeled SLN. The scale bar is 5 mm.

Figure S7. Primary skin irritation evaluation. (A) GERTs and control solution on the sterile gauze dressing. (B) Photographs of the dressing on the rabbit dorsum. (C to E) The skin reaction at different points after removing the dressing.

Figure S8. Intradermal reactivity test. (A) Polar extraction solution of GERTs and its corresponding control solution. (B) Non-polar extraction solution of GERTs and its corresponding control solution. (C to E) The skin reaction at different post-injection time points.

Figure S9. The lymphatic territories in (left) human being and (right) rabbit. Human: 1.

parotid; 2. frontal cervical;3. posterior cervical; 4. axillary; 5. retroperitoneal; 6. popliteal; 7. superficial inguinal; 8. subclavicular; Rabbit: 1. parotid; 2. mandibular lymph nodes; 3. cervical; 4. axillary; 5. lumbar; 6. popliteal; 7. inguinal; 8. lateral sacral.

Figure S10. The location of SLNs and 2nd LN among different species. In mice model: (a) Photograph of MB-labeled SLN and 2nd LN; (b) NIR fluorescence images of SLN and draining LNs; (c) NIR-IIb quantum dots imaging guided surgery of popliteal lymph node and lumbar LN. In rabbit model: (d) GERTs guided SLN tracking; (e) MB-labeled SLN and 2nd LN; (f) ICG-labeled SLN and 2nd LN.

Figure S11. Comparison of MB, ICG and GERTs for SLN positioning. (A) Photograph of nipple before tracer injection. (B) After tracer injection and 10 minutes vigorous massage at the injection site. (C) MB, ICG labeled lymphatic vessels, SLN, 2nd LN and GERTs labeled SLN.

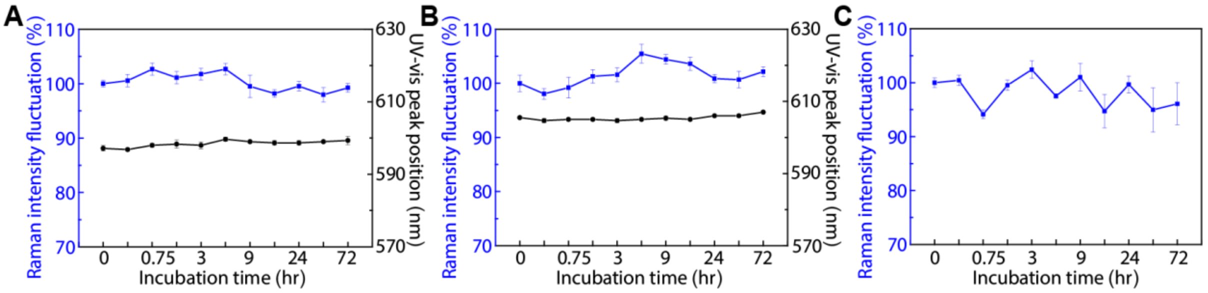

Figure S1. Stability measurements of UV-vis extinction spectra and Raman intensity of GERTs incubated with (A) 0.5% PVP, (B) 0.5% PVP-Saline and (C) 0.5% PVP-10%FBS. The extinction spectrum of GERTs in 10% FBS is not recorded since the absorption of FBS overlaps with the resonance peak of GERTs.

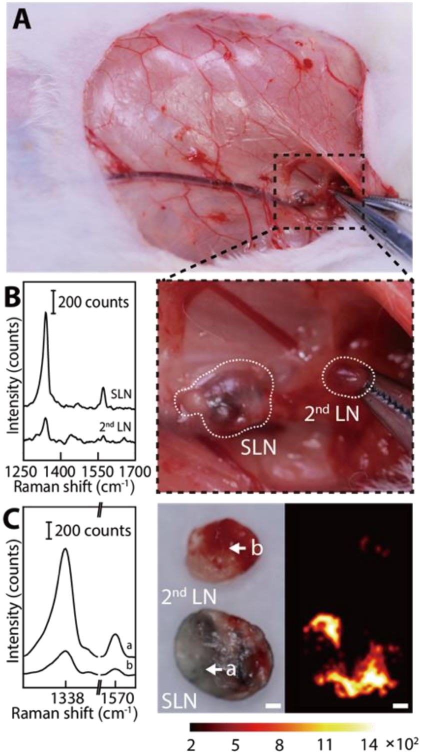

Figure S2. Evolution of *in vivo* LN tracing and *ex vivo* LN SERS imaging at post-injection 24 h of GERTs. (A) Photographs and enlarged images of SLN and 2nd LN at different post-injection time points of GERTs, and (B) the corresponding *in vivo* SERS spectra. (C) *Ex vivo* photographs, SERS images and the corresponding SERS spectra from the positions indicated on the dissected SLN (arrow a) and 2nd LN (arrow b). All Raman images are plotted using the Raman band at 1338 cm^{-1}. The scale bars are 400 μm.

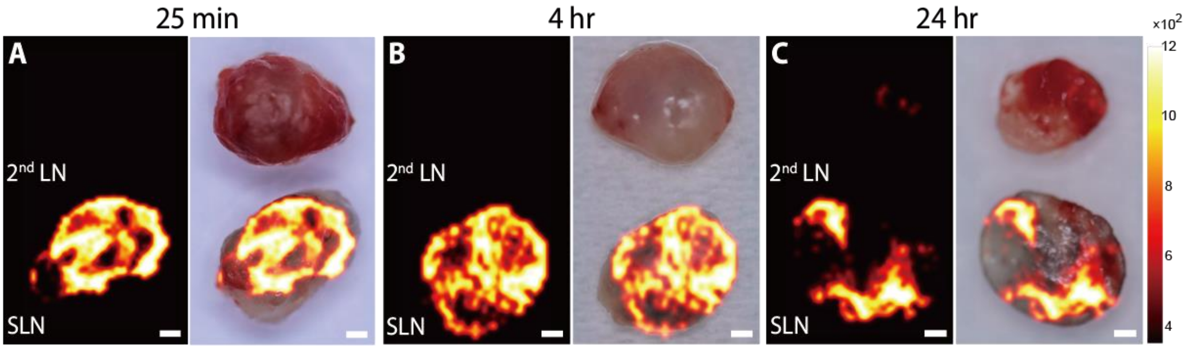

Figure S3. SERS and merge image of a dissected SLN and 2nd LN at different time points after GERTs injection. The scale bars are (A) 400 µm, (B) 750 µm and (C) 400 µm, respectively.

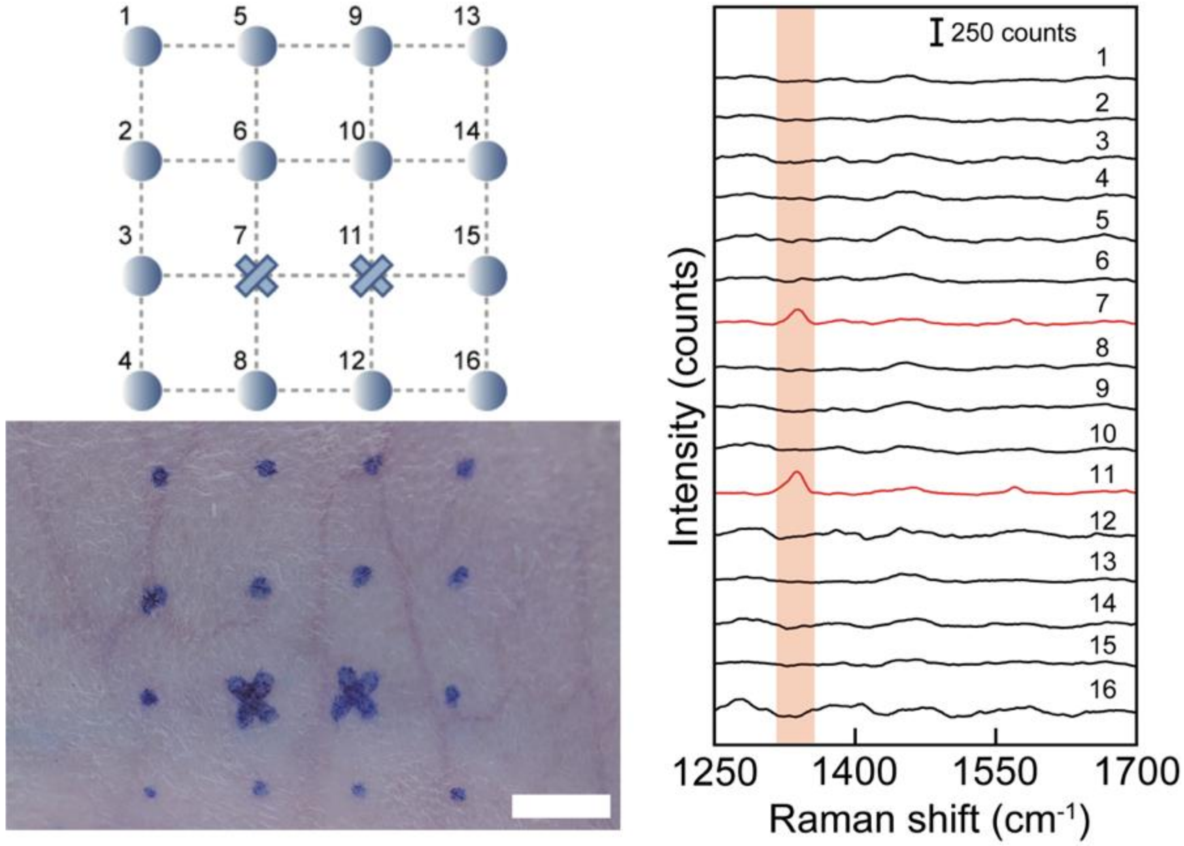

Figure S4. Total Raman spectra of 16 detection points by portable Raman scanner during SLN subcutaneous positioning. The acquisition parameter is 785 nm laser excitation, 400mW laser power density and 0.2s acquisition time. The scale bar is 5 mm.

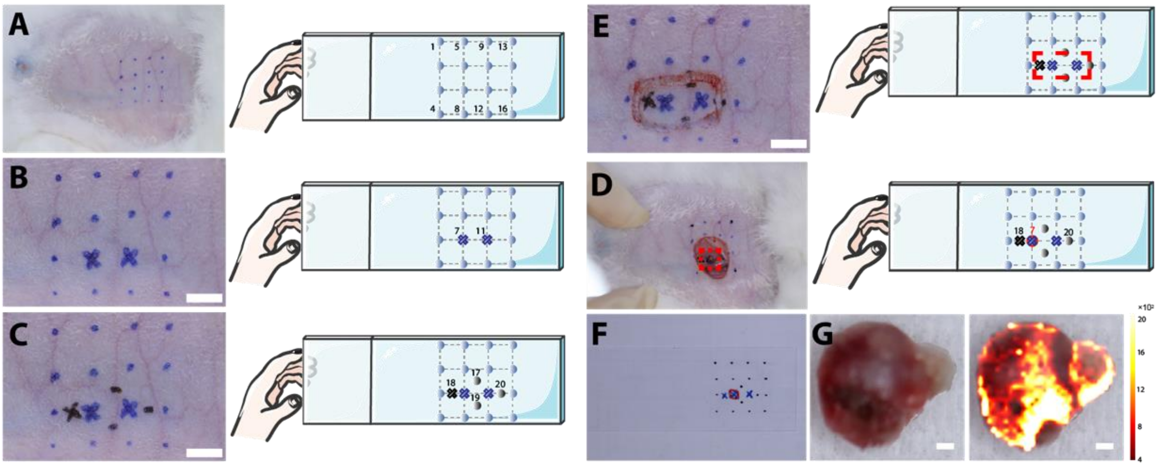

Figure S5. GERTs achieve accurate preoperative positioning of SLN. (A to D) The marks on the skin are drawn with the same size on the glass, which used to confirm the SLN position after dissecting the skin. (E) Placed the glass slide over the skin and match the dots on the glass with the dots on the skin one by one while skin dissected. The point 7 on the slide completely coincides with the position of SLN in vivo. (F) Marks on the glass slide. (G) Light image and merge image of dissected SLN by a Raman confocal system. All Raman images are plotted using the Raman band at 1338 cm $^{-1}$. The scale bar are (B, C, E) 5 mm and (G) 200 μm.

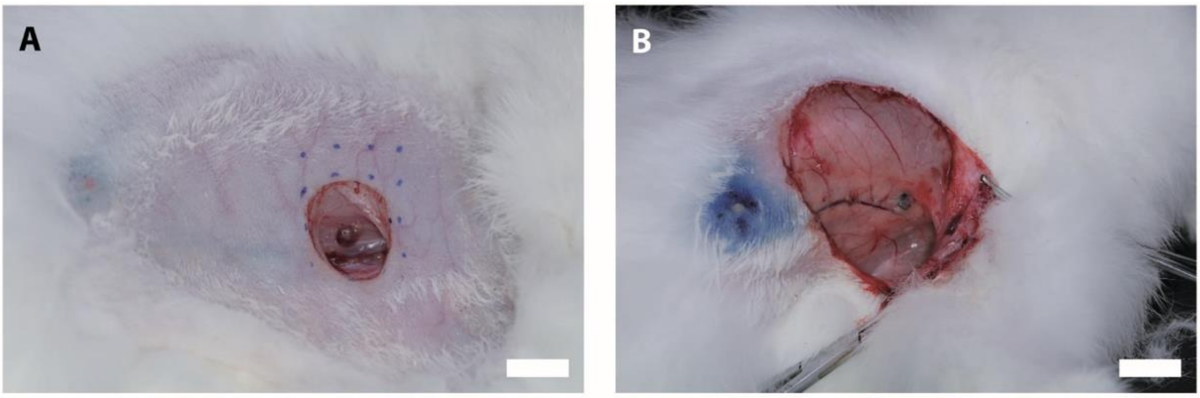

Figure S6. Comparison of surgical window size for SLN resection after GERTs (A) and MB (B) labeled SLN. The scale bar is 5 mm.

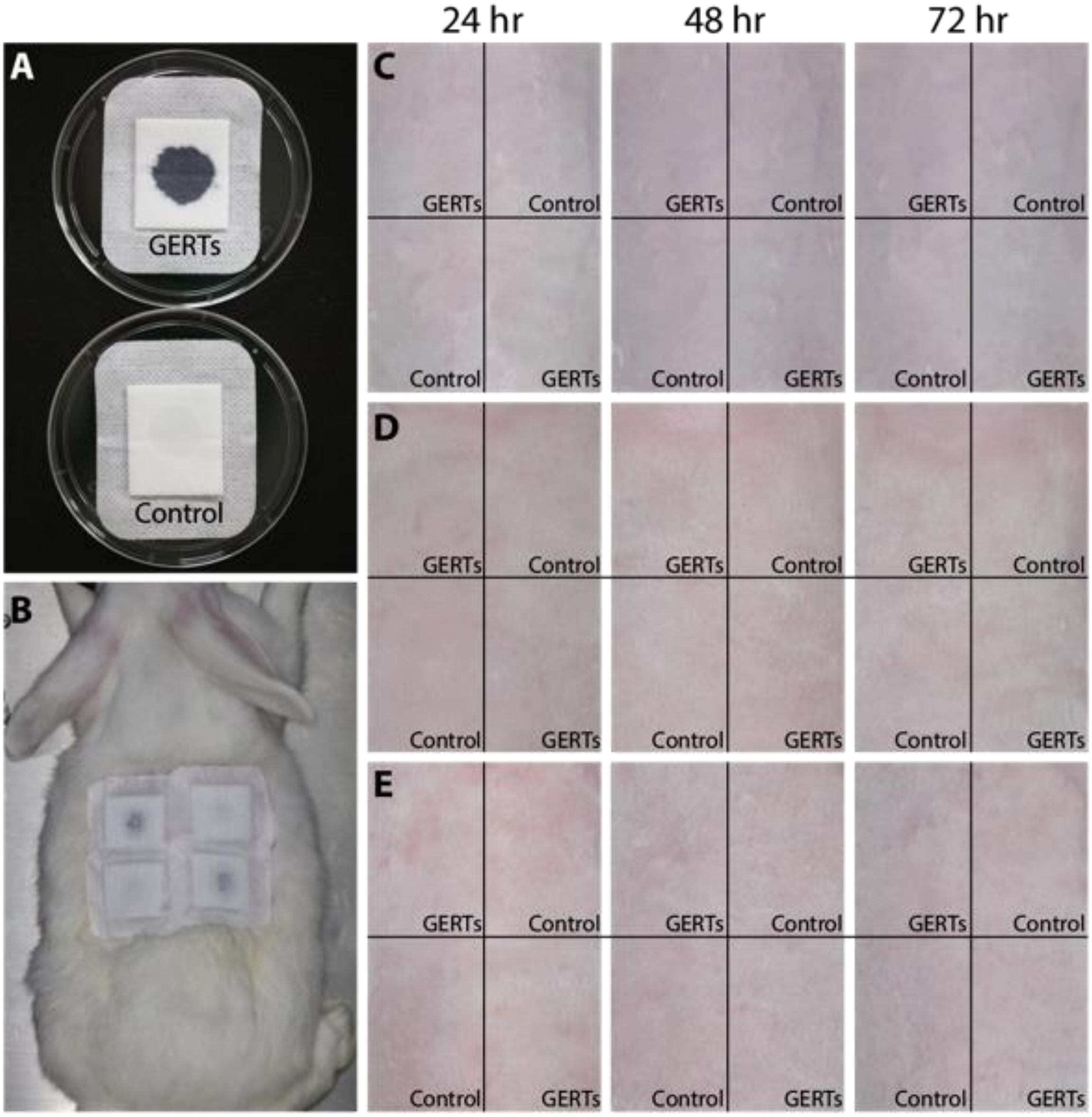

Figure S7. Primary skin irritation evaluation. (A) GERTs and control solution on the sterile gauze dressing. (B) Photographs of the dressing on the rabbit dorsum. (C to E) The skin reaction at different points after removing the dressing.

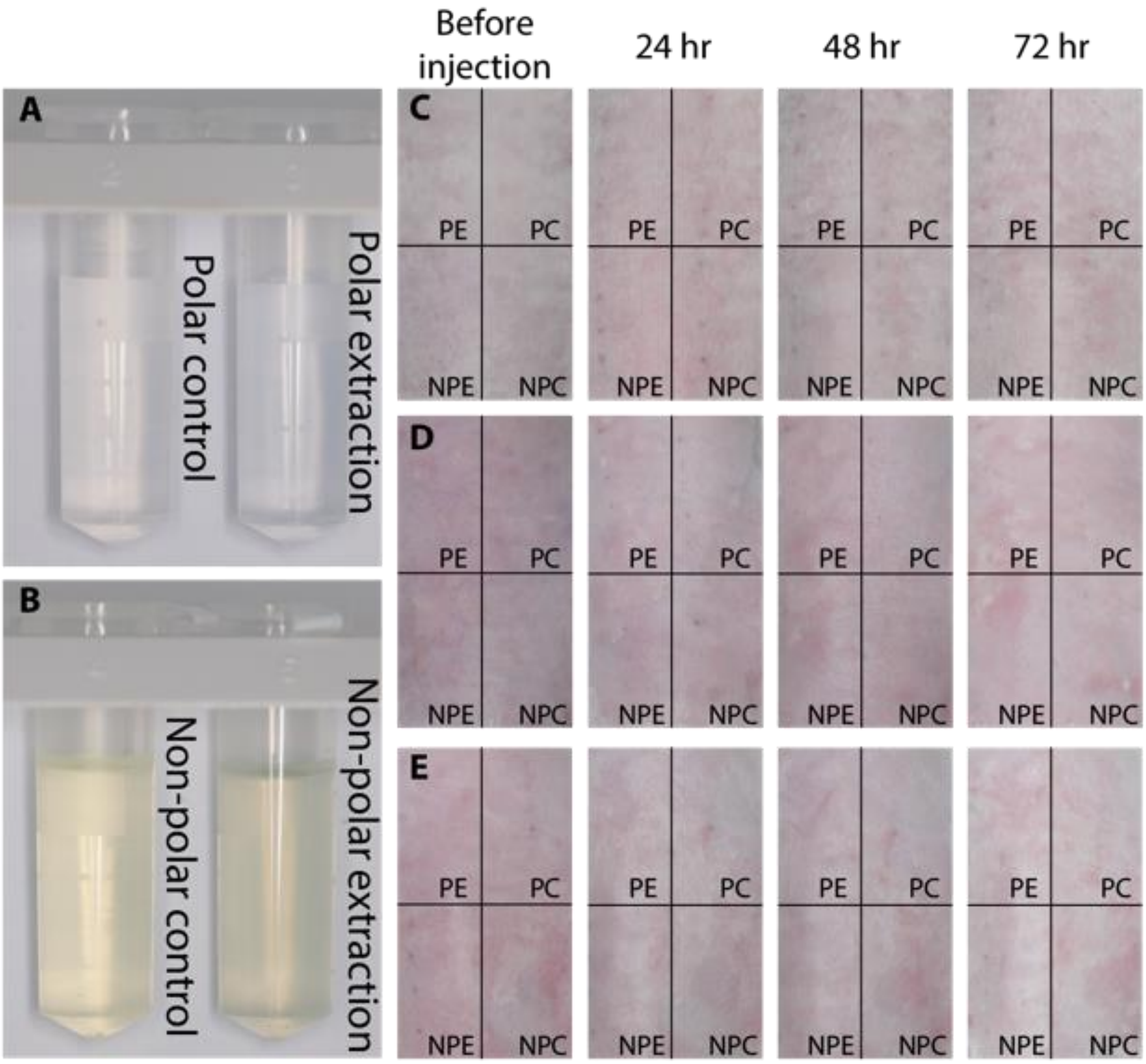

Figure S8. Intradermal reactivity test. (A) Polar extraction solution of GERTs and its corresponding control solution. (B) Non-polar extraction solution of GERTs and its corresponding control solution. (C to E) The skin reaction at different post-injection time points.

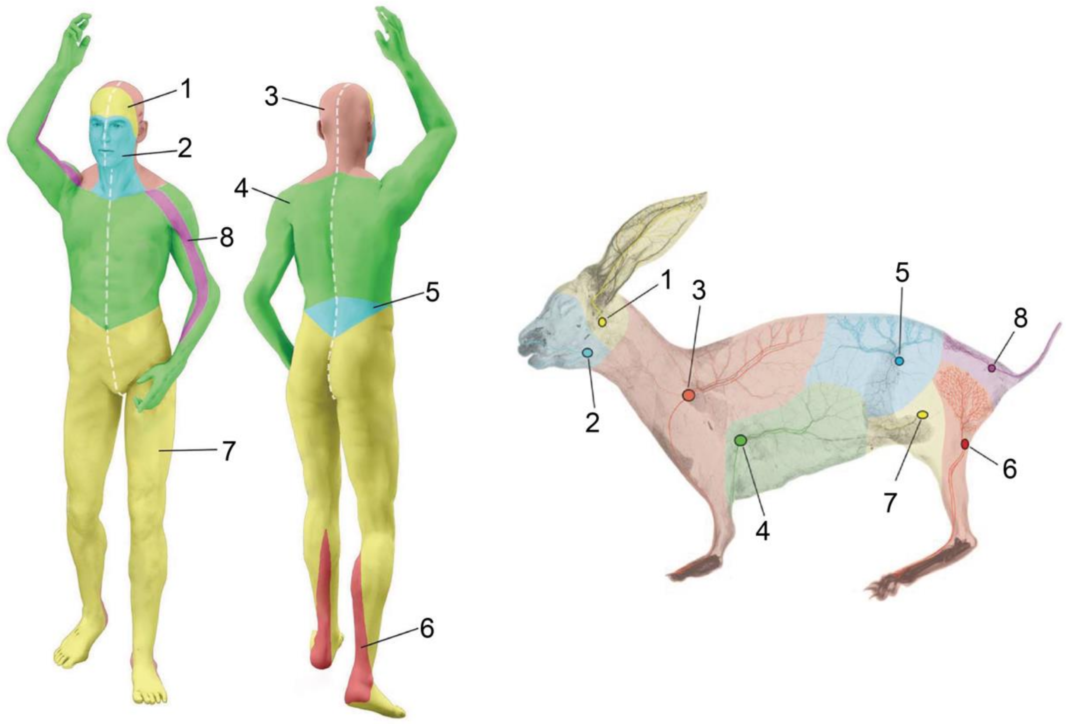

Figure S9. The lymphatic territories in (left) human being and (right) rabbit.[1] Human: 1. parotid; 2. frontal cervical;3. posterior cervical; 4. axillary; 5. retroperitoneal; 6. popliteal; 7. superficial inguinal; 8. subclavicular; Rabbit: 1. parotid; 2. mandibular lymph nodes; 3. cervical; 4. axillary; 5. lumbar; 6. popliteal; 7. inguinal; 8. lateral sacral.

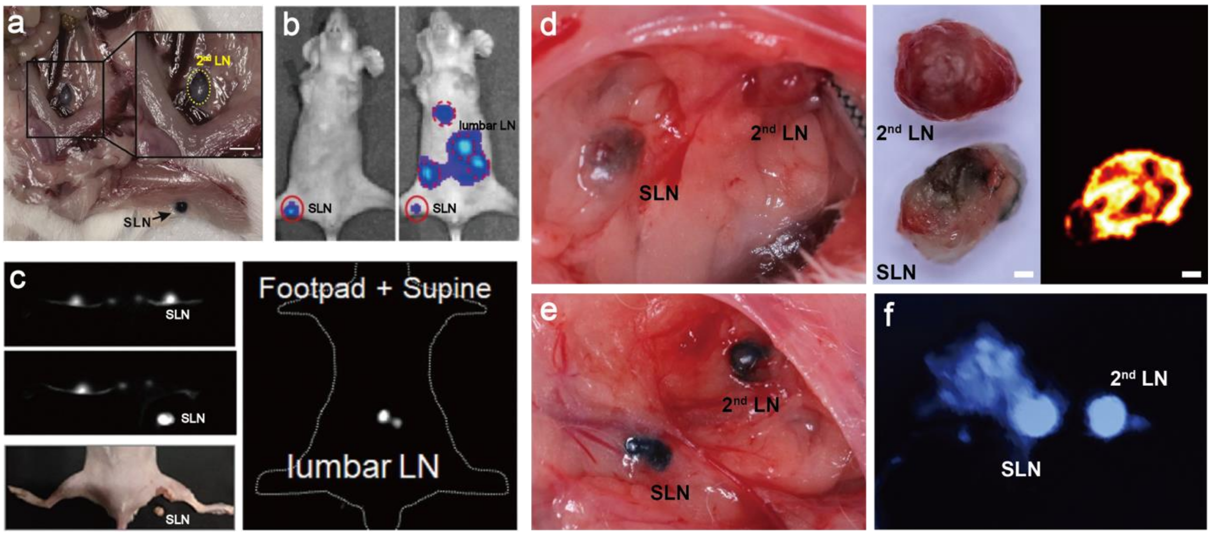

Figure S10. The location of SLNs and 2nd LN among different species. In mice model: (a) Photograph of MB-labeled SLN and 2nd LN[2]; (b) NIR fluorescence images of SLN and draining LNs[3]; (c) NIR-IIb quantum dots imaging guided surgery of popliteal lymph node and lumbar LN[4]. In rabbit model: (d) GERTs guided SLN tracking; (e) MB-labeled SLN and 2nd LN; (f) ICG-labeled SLN and 2nd LN.

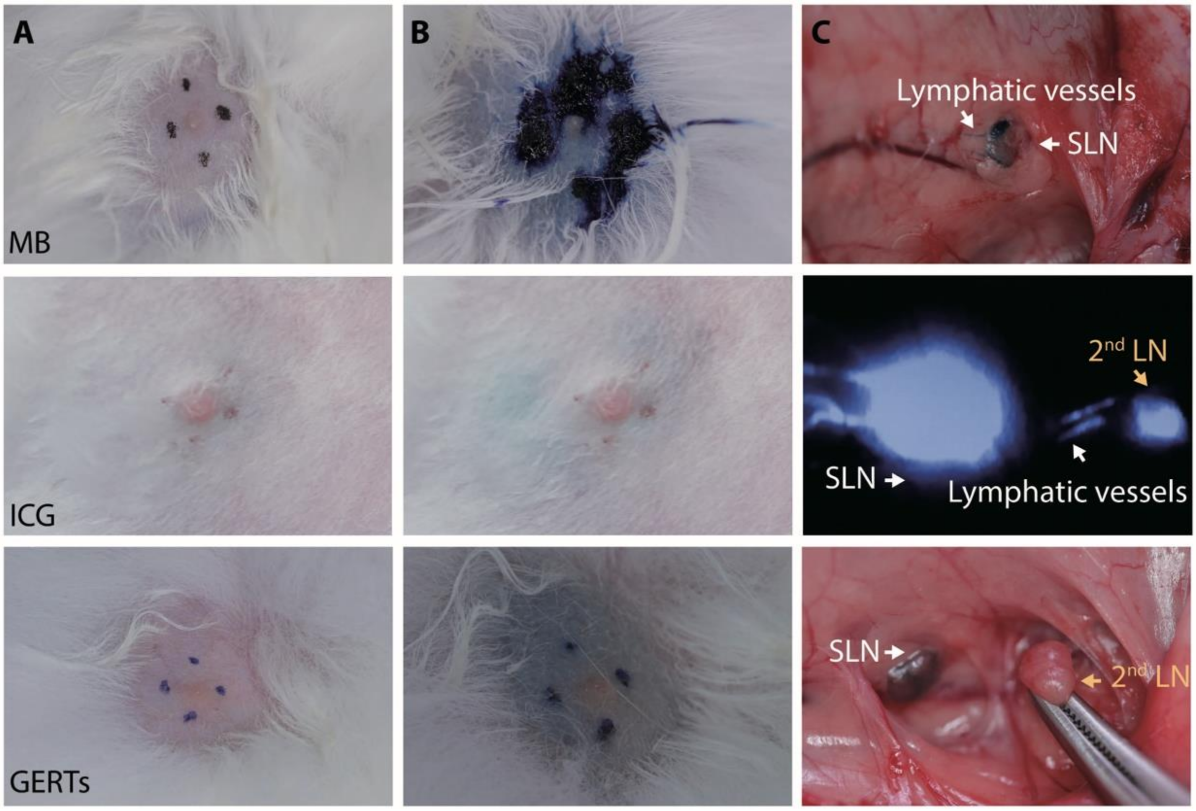

Figure S11. Comparison of MB, ICG and GERTs for SLN positioning. (A) Photograph of nipple before tracer injection. (B) After tracer injection and 10 minutes vigorous massage at the injection site. (C) MB, ICG labeled lymphatic vessels, SLN, 2nd LN and GERTs labeled SLN.

Reference:

[1] a) M. A. Soto-Miranda, H. Suami, D. W. Chang, *The Anatomical Record* **2013**, 296 (6), 965; b) H. Suami, S. Yamashita, M. A. Soto-Miranda, D. W. Chang, *PLoS One* **2013**, 8 (7), e69222.

[2] Z. Bao, Y. Zhang, Z. Tan, X. Yin, W. Di, J. Ye, *Biomaterials* **2018**, 163, 105, https://doi.org/10.1016/j.biomaterials.2018.02.020.

[3] R. Wei, G. Jiang, M. Lv, S. Tan, X. Wang, Y. Zhou, T. Cheng, X. Gao, X. Chen, W. Wang, *Theranostics* **2019**, 9 (24), 7325.

[4] R. Tian, H. Ma, S. Zhu, J. Lau, R. Ma, Y. Liu, L. Lin, S. Chandra, S. Wang, X. Zhu, *Adv. Mater.* **2020**, 32 (11), 1907365
